# Abscisic Acid Regulates the 3-Hydroxy-3-methylglutaryl CoA Reductase Gene Promoter and Ginsenoside Production in *Panax quinquefolium* Hairy Root Cultures

**DOI:** 10.3390/ijms20061310

**Published:** 2019-03-15

**Authors:** Ewa Kochan, Ewa Balcerczak, Piotr Szymczyk, Monika Sienkiewicz, Hanna Zielińska-Bliźniewska, Grażyna Szymańska

**Affiliations:** 1 Department of Pharmaceutical Biotechnology, Medical University of Lodz, Muszyńskiego l, 90-151 Lodz, Poland; piotr.szymczyk@umed.lodz.pl (P.S.); grazyna.szymanska@umed.lodz.pl (G.S.); 2 Laboratory of Molecular Diagnostics and Pharmacogenomics, Department of Pharmaceutical Biochemistry and Molecular Diagnostics, Interfaculty Cathedral of Laboratory and Molecular Diagnostics, Medical University of Lodz, Muszyńskiego 1, 90-151 Lodz, Poland; ewa.balcerczak@umed.lodz.pl; 3 Department of Allergology and Respiratory Rehabilitation, 2nd Chair of Otolaryngology, Medical University of Lodz, Żeligowskiego 7/9, 90-725, Lodz, Poland; monika.sienkiewicz@umed.lodz.pl (M.S.); anna.zielinska-blizniewska@umed.lodz.pl (H.Z.-B.)

**Keywords:** *3-hydroxy-3-methylglutaryl CoA reductase* gene promoter, ginsenosides, hairy roots, *Panax quinquefolium*

## Abstract

*Panax quinquefolium* hairy root cultures synthesize triterpenoid saponins named ginsenosides, that have multidirectional pharmacological activity. The first rate-limiting enzyme in the process of their biosynthesis is 3-hydroxy-3-methylglutaryl CoA reductase (HMGR). In this study, a 741 bp fragment of the *P. quinquefolium* *HMGR* gene (*PqHMGR*), consisting of a proximal promoter, 5′UTR (5′ untranslated region) and 5′CDS (coding DNA sequence) was isolated. In silico analysis of an isolated fragment indicated a lack of tandem repeats, miRNA binding sites, and CpG/CpNpG elements. However, the proximal promoter contained potential *cis*-elements involved in the response to light, salicylic, and abscisic acid (ABA) that was represented by the motif ABRE (TACGTG). The functional significance of ABA on *P. quinquefolium HMGR* gene expression was evaluated, carrying out quantitative RT-PCR experiments at different ABA concentrations (0.1, 0.25, 0.5, and 1 mg·L^−1^). Additionally, the effect of abscisic acid and its time exposure on biomass and ginsenoside level in *Panax quinquefolium* hairy root was examined. The saponin content was determined using HPLC. The 28 day elicitation period with 1 mg·L^−1^ ABA was the most efficient for Rg2 and Re (17.38 and 1.83 times increase, respectively) accumulation; however, the protopanaxadiol derivative content decreased in these conditions.

## 1. Introduction

The perennial herbs belonging to the *Panax* genus display multidirectional biological activity, and this has been attributed to their high ginsenoside content; these being triterpene saponins. Comprehensive investigations have determined that ginseng has many pharmacological effects on the immune, cardiovascular, endocrine, and central nervous systems [1]. The extracts isolated from *Panax ginseng* or *P. quinquefolium* (American ginseng) have been found to demonstrate anti-aging, anti-stress, anti-fatigue, and hepatoprotective properties [1,2,3].

Ginsenosides are classified into two groups depending on aglycone structure, namely dammarane type or oleanane type. The dammarane group, characterized by a tetracyclic skeleton, is home to most ginsenosides, including protopanaxadiol (PPD) and protopanaxatriol (PPT) derivatives. The structure of the PDD saponins is characterized by three features: glycosylation site(s) located at C3 and/or C20, a linear linkage between glycosyl chains, and acylation occurring at the 6-OH of the terminal glucose of a three-sugar chain; PPTs are characterized by glycosylation site(s) at C6 and/or C20, at the most two glycosyl chains, and a linear linkage between saccharide chains [4].

**Figure 1:**
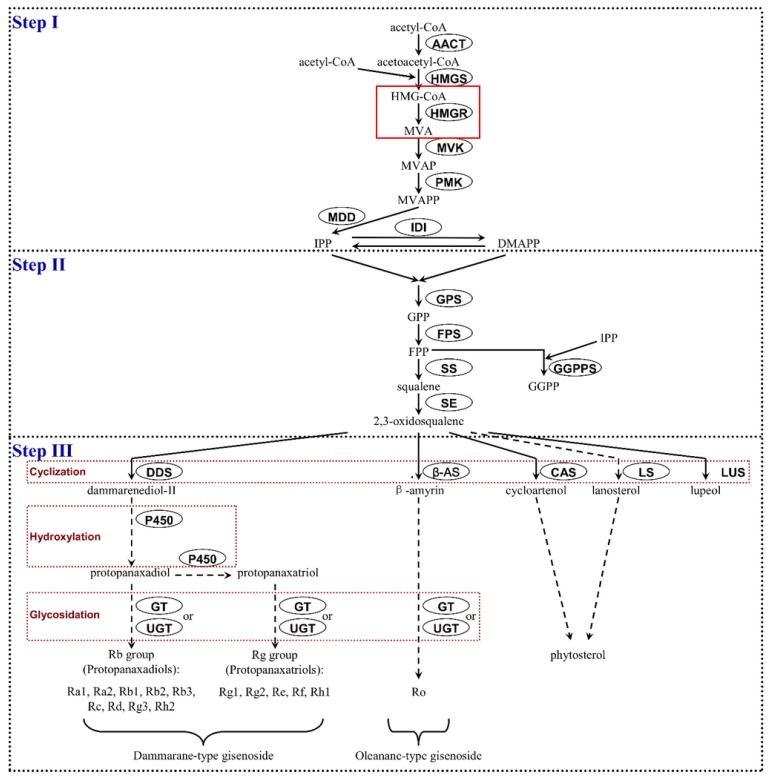
Biosynthetic pathway of ginsenosides according to [6]. AACT—acetyl-CoA acetyltransferase; HMGS—HMG-CoA synthase; HMGR—HMG-CoA reductase; MVA—mevalonate; MVK—mevalonate kinase; MVAP—mevalonate phosphate; PMK—phosphomevalonate kinase; MVAPP—mevalonate diphosphate; MDD—mevalonate-5-diphosphate decarboxylase; IDI—isopentenyl diphosphate isomerase; IPP—isopentenyl diphosphate, DMAPP—dimethylallyl diphosphate, GPS—geranyl diphosphate synthase; GPP geranyl diphosphate, FPS—farnesyl diphosphate synthase; FPP—farnesyl diphosphate, SS—squalene synthase; SE—squalene epoxidase, GGPPS—geranylgeranyl diphosphate synthase; GGPP—geranylgeranyl diphosphate; DDS—dammarenediol synthase; P450—cytochrome P450; GT—glycosyltransferase; UGT—UDP-glycosyltransferase, β-AS—beta-amyrin synthase; CAS—cycloartenol synthase; LS—lanosterol synthase; LUS—lupeol synthase. Solid red-frame means the reaction catalyzed by HMGR enzyme; Dotted red-frame—putative late steps of ginsenoside biosynthesis; dotted arrow—putative reaction steps leading to individual ginsenosides and phytosterol, solid arrows—well recognized reaction steps in ginsenoside biosynthesis pathway.

Ginsenosides are formed in the cytosol through the mevalonate (MVA) pathway involving more than 20 steps as shown in Figure 1, involving several enzymes, including 3-hydroxy-3-methylglutaryl coenzyme A reductase (HMGR), farnesyl pyrophosphate synthase (FPS), squalene synthase (SQE), dammarenediol-II synthase (DS), β-amyrin synthase (AS), cytochrome P450 (CYP450), and UDP-glycosyltransferase (UGT) [5].

The analysis of available literature indicated that 3-hydroxy-3-methylglutaryl coenzyme A reductase (HMGR) is considered to act as the first rate-limiting enzyme of the MVA pathway in plants [7,8]. This enzyme catalyzes the NAD(P)H-dependent reduction of HMG-CoA to mevalonate and can regulate ginsenoside biosynthesis by regulating the production of the ginsenoside precursors IPP (isopentenyl pyrophosphate) and DMAPP (dimethylallyl pyrophosphate) [5]. Sequencing of the *P. ginseng* genome suggests the presence of eight *HMGR* genes, four of which are similar to *PgHMGR1* and the other four are related to *PgHMGR2*. Further analysis of PgHMGR protein sequences showed two shorter subclasses related to *HMGR1* (1.1 and 1.2) and another two subclasses (2.1 and 2.2) built from longer HMGR forms similar to *PgHMGR2*. While *PgHMGR1* gene expression is relatively stable, *PgHMGR2* expression demonstrated greater variation among studied tissues. Only one of the eight *PgHMGR* genes, namely PG15732 from *PgHMGR_1.2_*, differed from the typical *PgHMGR* genomic organization based on four exons and three introns [9].

Studies of the *P. quinquefolium* transcriptome confirms the presence of several *HMGR* contigs, with five *HMGR* unique transcripts observed based on the Kyoto Encyclopedia of Genes and Genomes pathway assignment [10]. The presence of multiple *HMGR* genes is a common property of plants, increasing the complexity of *HMGR* expression regulation [11].

Abscisic acid (ABA), a carotenoid derivative synthesized de novo in plastids from the C_40_β-carotene molecule [12], is an important phytohormone controlling various physiological processes in plants, such as seed maturation and germination, dormancy, seedling growth, and stomatal behavior. It also regulates fruit-ripening and somatic embryogenesis [12,13,14]. In addition, ABA has been defined as a stress plant hormone because of its rapid accumulation in response to stress. ABA plays a central role in drought and salt-stress response in plants [15]. It has been noted that increased activation of some genes and the biosynthesis of specific proteins may improve the adaptation of the plant to drought or salt-stress conditions. For example, in *Panax* sp., the expression of S-adenosyl-L-methionine synthetase, PgWRKY1,2,4,5,8,9, LHT-type plant amino acid-transporter, and MYB transcription factor is activated in response to ABA treatment [16,17,18,19,20]. Literature data also indicates that abscisic acid can regulate the biosynthesis of secondary metabolites. Gupta et al. [21] and Hao et al. [22] noted a significant increase of tanshinone I content in the hairy roots of *Salvia miltiorrhiza* Bunge and of two salvianolic acids in *S. miltiorrhiza* Bge. f. alba grown under the influence of ABA.

The aim of the present study is to identify the proximal promoter of *P. quinquefolium HMGR* (*PqHMGR*) and characterize the potential *cis*-elements within it. A key novel aspect of the present study is that it examines the influence of ABA on *HMGR* gene expression and validates the obtained results by quantitative RT-PCR at various concentrations of ABA. In addition, it examines the effect of ABA on biomass and ginsenoside production in transformed *P. quinquefolium* root cultures. This is the first study to evaluate the influence of different concentrations of ABA on the content of eight triterpene saponins (Rb1, Rb2, Rb3, Rc, Rd, Rg1, Rg2, and Re) in flask-grown cultures. It also determines the optimal concentration and exposure time to ABA in this context.

## 2. Results

### 2.1. *In silico* Analysis of P. quinquefolium HMGR Promoter

**Figure 2:**
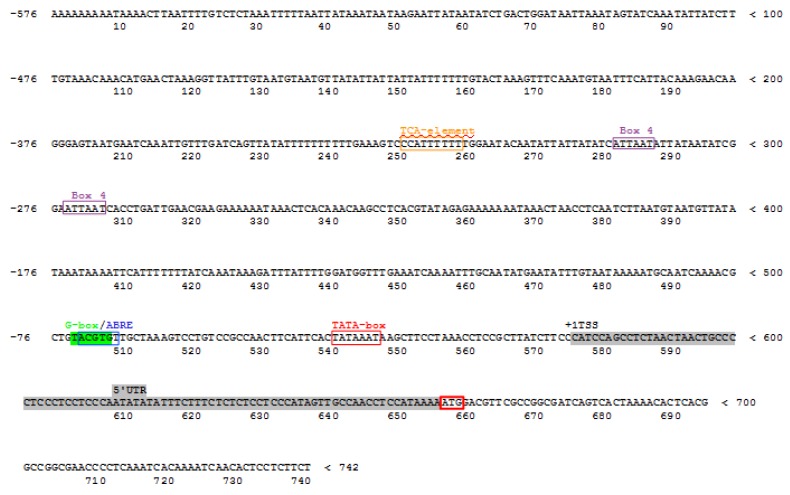
Important *cis*-regulatory elements found in *PqHMGR* promoter using the PlantCARE database. Expression of *PqHMGR* gene in hairy roots treated with abscisic acid. Different colors of frames mean different *cis*-active elements (orange frame: TCA *cis*-element- probably involved in the response to salicylic acid, purple frame: Box 4 *cis*-element involved in the response to light, green frame: G -box *cis*-element involved in the response to light, blue frame: ABRE *cis*-element involved in the response to abscisic acid, red frame containing TATAAAT sequence means TATA-box. Red frame containing ATG sequence is the translation initiation site. Grey color indicates the 5′UTR sequence.

The 741 bp genomic DNA fragment of the *3-hydroxy-3-methylglutaryl CoA reductase* gene was isolated from hairy root cultures of *P. quinquefolium* and has been deposited at GenBank under accession No KT869137.1. This region encompasses the proximal promoter, the 5′UTR and 5′CDS fragment of the coding DNA sequence (CDS) of the *HMGR* gene. The section 5′UTR located between 577 and 657bp undergoes transcription; however, it is not involved in the translation process. The 576 bp proximal promoter region was found to contain the TATA box between −35 and −28 bp in relation to the potential transcription start site (TSS). The prediction of TATA-box and transcription initiation site (TIS) location was performed as described in Materials and Methods. Some features characteristic of promoters, such as tandem repeats, CpG/CpNpG islands, and miRNA target sequences, were not found anywhere in the isolated fragment; however, the potential *cis*-acting, 5–9 bp-long regulatory elements were localized in the proximal promoter of the *PqHMGR* gene, as shown in Figure 2. These potential *cis*-regulatory elements are homologous to the *cis*-acting elements of other plants, such as *Arabidopsis thaliana*, *Petroselinum crispum*, and *Nicotiana tabacum.* The following motifs were discovered: ABRE (TACGTG)—localized in the promotor region from −72 to −67 bp in relation to TSS and involved in modulating the response to abscisic acid; two other *cis*-active elements: BOX 4 (ATTAAT) and G-BOX (TACGTG), which may participate in response to light; and a TCA-element which is probably involved in the response to salicylic acid, as shown in Figure 2. These elements are grouped between −320 and −268 bp. The presence of ABRE motifs prompted the research into the impact of ABA on growth and ginsenoside yield in hairy root cultures of *P. quinquefolium.* In addition, the expression of *PqHMGR* gene was evaluated in control and in hairy root cultures treated with ABA.

**Figure 3:**
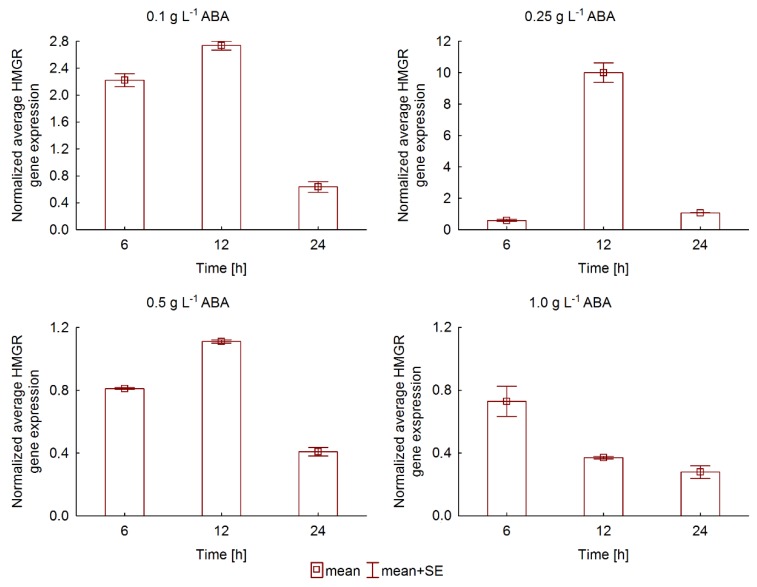
Normalized expression of *PqHMGR* gene in abscisic acid (ABA)-elicited *Panax quinquefolium* hairy roots.

RT-PCR analysis demonstrated temporal and concentration-dependent changes in *PqHMGR* gene expression as result of ABA treatment. The expression rate of *PqHMGR* clearly depends on abscisic acid concentration and exposure time, as shown in Figure 3. In general, the response to ABA treatment occurred quickly: an increase of *PqHMGR* gene transcription was observed just six hours after treatment with 0.1 mg·L^−1^ ABA.

The highest rate was noticed after twelve hours for the most ABA concentrations, except 1 mgL^−1^ ABA. Prolonged ABA action inhibited gene transcription. This relationship was observed for all the tested concentrations of abscisic acid. The normalized average *PqHMGR* gene expression demonstrated the highest mean induction rate (9.4-fold) at 0.25 mg·L^−1^ concentration of ABA after a twelve-hour treatment. Higher abscisic acid concentrations resulted in levels of *PqHMGR* transcription comparable to or lower than control samples.

### 2.2. The Effect of ABA Elicitation on Hairy Roots of P. quinquefolium Growth

**Figure 4:**
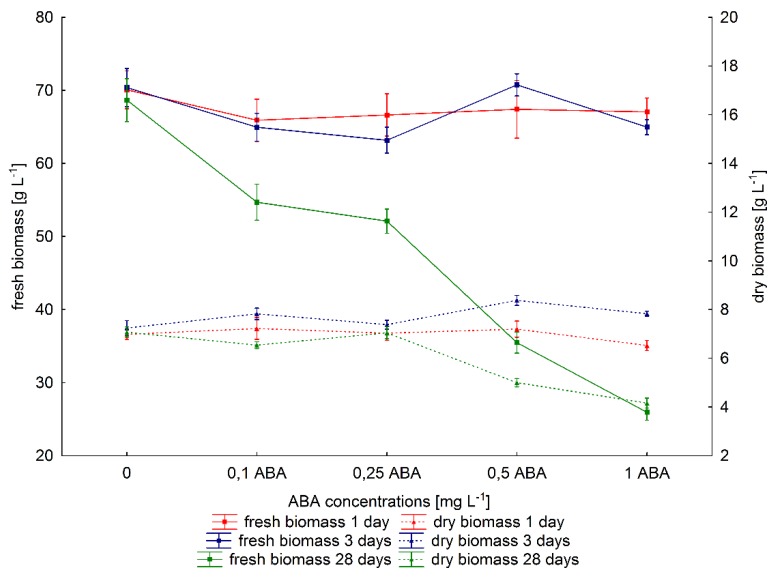
The effect of ABA on biomass of hairy roots of *P. quinquefolium.*

The effect of ABA on growth of *P. quinquefolium* hairy root cultures was estimated, as shown in Figure 4. No statistically significant differences in fresh biomass were observed after one- and three-day elicitation in comparison to control samples, regardless of ABA concentration (Kruskal–Wallis test). Although similar results were obtained for dry biomass after a one-day ABA treatment, slight increases in dry biomass were observed after a three-day exposure at higher ABA concentrations (0.5 and 1 mg·L^−1^). Completely different results were observed when abscisic acid was added on the day of inoculation and elicitation process lasted 28 days: in this case, both fresh and dry biomass were found to decrease in relation to untreated cultures. The use of 0.1 or 0.25 mg·L^−1^ ABA resulted in a decrease of fresh biomass of more than 20%, meanwhile dry biomass was similar to control values. Higher levels of ABA (0.5 and 1 mg·L^−1^) significantly inhibited growth, with fresh biomass decreasing by about 48% (0.5 mg·L^−1^ ABA) and 62% (1mg·L^−1^ ABA) in comparison to controls. The use of these ABA concentrations also resulted in a 30% and 41% decrease in dry biomass.

### 2.3. The Effect of ABA Elicitation on Ginsenoside Production in Hairy Roots of P. quinquefolium

**Figure 5:**
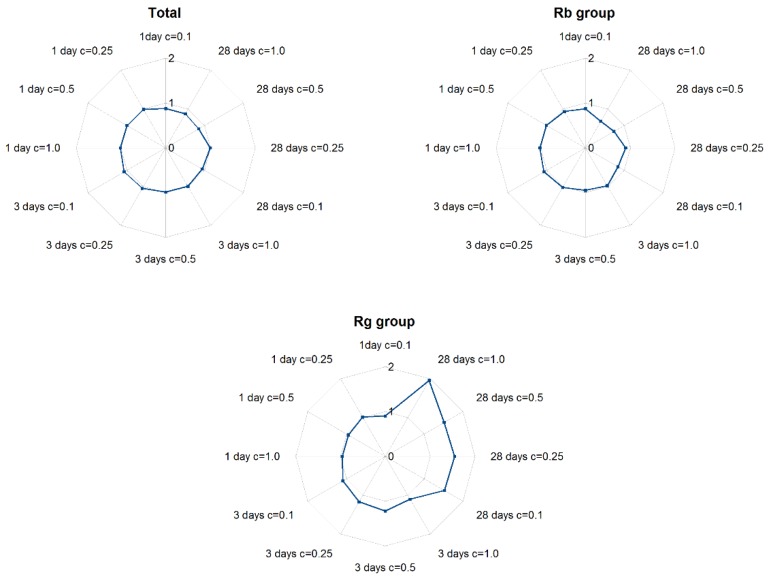
The effect of ABA elicitation on ginsenoside accumulation in hairy roots of *P. quinquefolium* cultivated in shake flasks. C- means ABA concentration.

The analysis of total content of the all examined metabolites (expressed as sum of Rb1, Rb2, Rb3, Rc, Rd, Rg1, Rg2, Re) showed that shorter exposure (one and three days) on ABA influenced saponin levels by a very small extent ranging between 13.51 and 14.39 mgg^−1^ dry weight (d.w.) and did not significantly differ from controls or each other. Similar observations were noted for lower concentrations of ABA (0.1 and 0.25 mg·L^−1^) for a 28 day elicitation. However, the application of 0.5 and 1 mg·L^−1^ ABA on the day of inoculation reduced total ginsenoside content by about 14–16.25%, as shown in Figure 5.

Protopanaxadiol derivatives (Rb-group saponins, including ginsenosides Rb1, Rb2, Rb3, Rc, and Rd together), demonstrated comparable, or slightly lower, levels than controls after one- and three-day elicitation. After 28 day exposure to ABA, an intense fall in their content was observed, resulting in the lowest amounts of Rb group ginsenosides: 7.97 mgg^−1^ d.w.—for 1 mg·L^−1^ ABA; these levels were 1.48-fold lower than in untreated samples. In addition, a greater decrease in the concentration of Rb group members was observed at higher ABA concentrations for the longest (28 day) elicitation. In contrast, protopanaxatriol derivative (Rg-group saponins, including Rg1, Rg2, and Re) content gradually increased with ABA concentration during the 28 day elicitation. The maximum yield of Rg group saponins was noted for 1 mg·L^−1^ ABA, with the level of protopanaxatriol derivatives being 1.9 times higher than controls.

One- and three-day exposure to ABA did not influence the protopanaxadiol or protopanaxatriol-derivative contents, irrespective of ABA concentration, and their levels were comparable to those of controls. The longest period of ABA elicitation caused a decrease in Rb group saponin level and an increase of Rg group level in relation to untreated samples. This trend was most noticeable for 1 mg·L^−1^ ABA.

**Figure 6:**
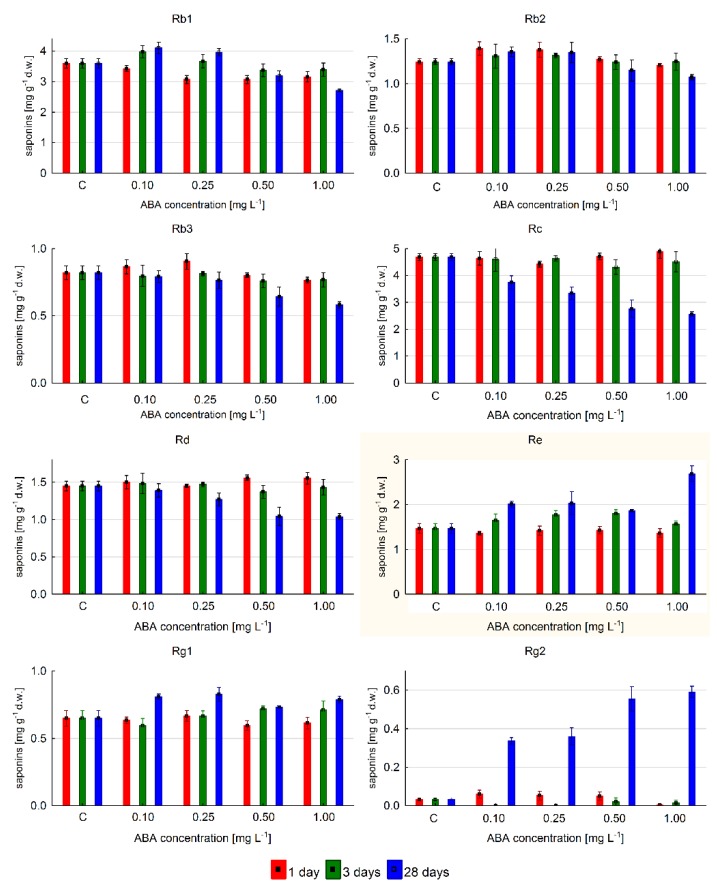
The accumulation of individual ginsenosides depending on ABA concentration in *P. quinquefolium* hairy root cultures growing in shake flasks.

The level of individual ginsenosides changed depending on ABA concentrations and time of exposure, as shown in Figure 6. The highest content was 4.9 mgg^−1^ d.w., determined for ginsenoside Rc after one-day elicitation with 1 mg·L^−1^ of ABA; however, this only reflected a 4% increase of Rc level in relation to control. Prolongation of ABA exposure time to three days did not cause significant changes in content, while 28 day elicitation decreased it significantly to below control levels. Similar changes in content were observed for ginsenoside Rd. Saponin Rb1 reached its maximum amount (13% higher than control) during the 28 day elicitation with 0.1 mg·L^−1^ ABA. The highest content of Rb2 and Rb3 metabolites was detected after 24 h exposure to 0.1 and 0.25 mg·L^−1^ ABA concentration, respectively. This represented a 10–12% increase of these saponin contents.

Obtained results indicated that all studied protopanaxatriol derivatives were found. The 28 day elicitation was the most efficient for Rg2 accumulation. Their level was 17.38 times higher than that of controls when 1 mg·L^−1^ ABA was used. The highest Re content (1.83-fold increase in relation to untreated samples) was associated with the longest exposure and the highest concentration of ABA. This compound dominated quantitatively. The Rg1 level slightly changed and was similar to control, irrespective of ABA concentration during shorter exposures (one and three days); however, the Rg1 level increased by approximately 27% at 0.1 and 0.25 mg·L^−1^ ABA as compared to untreated samples after 28 day exposure.

## 3. Discussion

The present study characterized the promoter of the gene encoding the *Pq*HMGR enzyme by the in silico method, using the PlantPAN and PlantCARE databases [23,24,25]. The length of the isolated DNA fragment is 575 bp, suggesting that it is a proximal promoter. Based on data available for *A. thaliana*, the complete plant promoter fragments are approximately 1113–1672 bp long [26]. In addition, no CpG sequence clusters were detected in the studied promoter region, implying a potential lack of inhibition of gene expression due to cytosine methylation; however, plant methyltransferases are able to modify not only CpG sequences but also CHH or CHG, and the lack of CpG does not exclude the epigenetic regulation of the *PqHMGR* gene [27]. For example, methyltransferase 1 and chromomethylase 3 are required for chromomethylase 2-mediated modification of a specific subset of CHH sequences. Also, the coordinated action of transcriptional silencer MOM1 and MORC1 is required for efficient targeting of a small subset of non-CG sequences by RNA-directed DNA methylation machinery [28]. Moreover, the lack of a CpG cluster close to the 5′ end of the gene suggests a lack of broad gene expression across the entire plant and that it may have a propensity to tissue-specific transcription [29]. It should be stressed that even the complete lack of DNA methylation observed in genes localized inside the DNA methylation valleys could not exclude the possibility of precise gene expression regulation by the selective action of transcription factors or chromatin epigenetic events [30].

The *PqHMGR* promoter does not contain tandem repeats, being the unstable regions commonly found in the genome. As many as 25% of all gene promoters contain such elements. Most often, their length does not exceed nine nucleotides and are referred to as *short tandem repeats* (STRs), *simple repeated sequences* (SSRs), or microsatellites. Tandem repeats are associated with a higher frequency of mutation, affecting their DNA sequence composition and length, typically leading to changes in nucleosome-free organization of the promoter region [31,32,33]. Such changes of chromatin structure in promoter regions results in a higher divergence of the gene expression rate [31,34,35]. Assuming the lack of tandem repeats, these effects are unlikely to occur in the *PqHMGR* promoter.

In silico analysis allowed the location of potential *cis*-factors to be identified within the promoter of the gene encoding HMG-CoA reductase, which showed homology with similar factors found in other plant species. The presence of G-box and Box 4 elements in the *PqHMGR* promoter, possibly participating in the light response, has been confirmed. The light-responsive *cis*-elements have previously been found in promoters of *HMGR* genes from tomato, maidenhair tree (*Gingko biloba*), mulberry (*Morus alba*), kutki/kutaki (*Picrorhiza kurroa*), red sage (*Salvia miltiorrhiza*), and *Andrographis paniculata* [36,37,38,39,40,41]. The presence of a G-BOX element suggests that the isolated *PqHMGR* promoter may be homologous to *PgHMGR1*. This element was not observed in the *PgHMGR2* promoter [7].

Also, the presence of *cis*-active elements responsive to salicylic acid suggests that the *PqHMGR* gene may play a role in plant-defense or development-regulation mechanisms [42] (Ma et al. 2018). These sequences are rather a common property of the plant *HMGR* gene regulatory sequences studied in *Gingko biloba*, *Michelia chapensis*, *Salvia miltiorrhiza*, *Malus domestica,* and tobacco [39,41,43,44,45,46].

Our bioinformatic searches indicated also the existence of the ABRE factor of the TACGTG sequence in the *PqHMGR* promoter. An identical fragment was found in *Arabidopsis thaliana*, *Vitis pseudoreticulata*, *Vitis vinifera cv*. *Carignan*, and *V. vinifera cv*. *Thompson Seedless.* According to the literature data, this *cis*-element is known to mediate the response to the action of ABA [47,48,49]. Abscisic acid is known to participate in regulating the plant response to drought and dehydration stress [15]. In these conditions, the plant membrane lipids disintegrate, leading to the destruction of cell membranes [50]. Among the major components of plant lipid membrane mediating their functionality are phytosterols; these are produced mainly by the mevalonic pathway, which also facilitates ginsenoside biosynthesis, as shown in Figure 1. The crucial enzyme precisely controlling the efficiency of the entire pathway is HMGR [7]. Previous studies on plant *HMGR* genes have highlighted a moderate response to ABA treatment [41,51]. A higher induction rate, approximately 3- to 4-fold, was observed for *HMGR* from mulberry (*Morus alba*) [37]; however, our results show even a 9.4-fold increase of *PqHMGR* expression after ABA treatment. This observation suggests abscisic acid may play an important role in the regulation of *PqHMGR* gene activity, independent of its product level. It is also probably that *PqHMGR* gene activity can influence the regulation of ginseng saponin biosynthesis especially that a previous investigation found the significant correlation of *HMGR* gene expressions with total ginsenoside [52]. In addition, Kim et al. [7] indicate that the reduction of its expression could reduce the level of ginseng saponins. Our results demonstrated that different concentrations of exogenous ABA and exposure times affect individual ginsenoside accumulation in different ways.

The above findings, both ours and those of other authors [7,37,52], indicate that ABA can be an important signal molecule involved in regulation of plant secondary metabolite production. This concerns not only the influence on the biosynthesis of ginseng saponins but also other biologically-active compounds. Exogenous abscisic acid enhances salvianolic acid content in hairy root cultures of *Salvia miltiorrhiza* Bge. f. alba [22] and paclitaxel production in *Taxus chinensis* suspension cell cultures [53]. In contrast, Sun et al. [54] report a significant decrease in the levels of shikonin and its derivatives in cell cultures of *Onosma paniculatum*. Furthermore, the addition of ABA to these cultures caused a decrease in the activities of phenylalanine ammonia-lyse and *p*-hydroxybenzoic acid-geranyltransferase: key enzymes involved in shikonin biosynthesis [54]. Abscisic acid stimulated terpene production and decreased petunidin-3-G content, but not the levels of other anthocyanins, in *Vitis vinifera* L.cv Malbec berries; however, it elicited the synthesis of anthocyanins and mono- and sesquiterpenes and decreased the levels of membrane sterols in the leaves [55]. In strawberry leaves, abscisic acid stimulated phenolic acid production at a level proportional to ABA concentration [56].

Our findings indicate that exogenous supplementation of ABA can influence the growth and biomass of hairy roots of American ginseng. Shorter exposure (one and three days) to ABA did not influence fresh biomass, irrespective of its concentration. A small increase in dry biomass was observed after three days of elicitation by 0.5 and 1 mg·L^−1^ of ABA. Longer treatment with 0.5 and 1 mg·L^−1^ ABA significantly inhibited fresh biomass and dry biomass. Similar observations have been noted for *Salvia miltiorrhiza* Bge. f. alba transformed root [22] and *Onosma paniculatum* suspension cultures [54]. Other investigations have indicated that low concentrations of ABA can stimulate root growth while high ABA levels inhibit plant growth [57,58]. Effect of ABA on growth has also been found to depend on time of exposure to abscisic acid. The biomass of *Genista tinctoria* hairy root cultures was strongly inhibited when ABA was added to medium on the day of inoculation [59]. This observation is consistent with our results.

## 4. Materials and Methods

### 4.1. Plant Material

Hairy root cultures of *P. quinquefolium* were grown in 80 mL of modified B-5 medium without hormone addition [60]. The average inoculum size was about 317 mg of fresh weight (f.w.) and 31.7 mg of dry weight (d.w.). The cultures were maintained in the dark at 26 °C degrees on a rotary shaker (100 rpm).

### 4.2. Measurement of Hairy Root Culture Growth

Fresh weight of the material, after separating tissue from the medium, was weighed and recorded. For dry weight assessment, the tissue was dried at 100 °C for one hour, and then at 80 °C for 24 h. The results were expressed in g·L^−1^.

### 4.3. Isolation of Genomic DNA

The genomic DNA was isolated from 14 day old hairy roots of *P. quinquefolium* according to Khan et al. [61]. Its purity and concentration were determined using a Nanophotometer UV/VIS spectrophotometer (Implen, Munchen, Germany), based on its A260/280 and A260/230 ratio. Genomic DNA was used as a template DNA for the PCR reaction to isolate the *PqHMGR* promoter.

### 4.4. Promoter Isolation

A GenomeWalker™ Universal Kit (Takara Bio, Mountain View, CA, USA) was applied to isolate the potential promoter region of the *HMGR* gene of *P. quinquefolium* hairy roots according to the manufacturer’s instructions [62]. Two gene-specific primers, *GSP1* (5′AAT GGG AGA GGA AGA GCA TCA GAG GCT T3′) and *GSP2* (5′AGA AGA GGA GTG TTG ATT TTG TGA TTT GAG G3′), were used for the PCR reaction. These primers were designed based on a 5′ terminal fragment of the cDNA sequence (GenBank accession number: FJ755158.2) of the 3-hydroxy-3-methylglutaryl CoA reductase gene using the OligoCalc server [63].

The OligoCalc server was also applied to calculate the temperature of primer melting (T_M_). Based on our experience, the optimal T_M_ values resulting in a good quality PCR reaction are obtained by the salt adjusted (50 mM NaCl) algorithm [63].

Annealing temperature of primers was usually established within the range 2–4 °C below the T_M_ (temperature of melting). The PCR reaction was carried out using Advantage^®^ 2 Polymerase Mix (Takara Bio, Mountain View, CA, USA). The first part of the reaction consisted of the following elements: initial DNA denaturation (95 °C, time: 3 min), denaturation (95 °C, time: 30 s), annealing and extension (72 °C, time: 3 min), repeated in seven cycles. Following this, 32 cycles of denaturation (95 °C, time: 30 s) and annealing and extension (70 °C, time: 3 min) were performed. The final additional cycle was carried out at 72 °C for seven minutes. The nested PCR reaction was performed as follows: initial DNA denaturation (95 °C, time: 3 min), denaturation (95 °C, time: 30 s), annealing and extension (72 °C, time: 3 min), repeated in five cycles, followed by denaturation (95 °C, time: 30 s) and annealing and extension (67 °C, time: 3 min), for 20 cycles. The final additional cycle was carried out at 72 °C for seven minutes.

### 4.5. Cloning and Sequencing of the PqHMGR Gene Promoter

The amplified DNA fragments were TOPO-TA cloned into a pCR^®^IITOPO^®^ vector (Thermo Fisher Scientific, Waltham, MA, USA). Highly-efficient chemically-competent *Escherichia coli* DH5 alpha cells (New England Biolabs, Ipswich, MA, USA) were subjected to thermal shock and transformed by 5 μL of TOPO-TA cloning reaction product. The transformants were selected on Luria-Bertani medium (LB)-agar (2%) plates containing 30 μg·mL^−1^ kanamycin sulphate (Genos, Lodz, Poland). The plasmid DNA was isolated from liquid *E.coli* cultures growing on LB medium in the presence of 30 μg·mL^−1^ kanamycin sulphate [64]. The presence of an insert was verified by EcoRI digestion and subsequent 1% agarose gel electrophoresis. The selected clone was sequenced using a 3130xl Genetic Analyzer (Applied Biosystems, Waltham, MA, USA) at the CoreLab facility (Medical University of Lodz, Lodz, Poland). The sequencing process used M13 reverse and M13 forward primers possessing binding sites in the pCR^®^IITOPO^®^ vector.

### 4.6. In Silico Analysis of the P. quinquefolium HMGR Promoter

The studied sequence of the *PqHMGR* promoter was examined for the presence of tandem repeats, CpG/CpNpG islands, and microRNA (miRNA) binding sites using PlantPAN and PlantPAN 2.0 [65,66]. In addition, the presence of potential *cis* elements was determined using PlantCARE (http://bioinfomatics.psb.ugent.be/webtools/plantcare/html) [25].

TATA-box and transcription initiation site (TIS) localization were established applying TSSP software and RegSite Plant DB (Softberry Inc., Mount Kisco, NY, USA) [23,67].

### 4.7. RNA Isolation and Reverse Transcriptase (RT)-PCR Analysis

RNA was isolated from hairy roots of *P. quinquefolium* after 6, 12, and 24 h elicitation with ABA by Total RNA Prep Plus Minicolumn Kit (A&A Biotechnology, Gdynia, Poland) according to the manufacturer’s instructions. The obtained samples of RNA were digested by RNase-free DNaseI (5 U/sample) to assure the complete removal of genomic DNA. The isolated RNA had an A260/280 ratio of 1.6–1.8. UV absorbance was used to determine the amount of RNA used in the cDNA reverse transcription reaction. The RNA concentration was set at 0.003 μg μL^−1^ in a final volume of 20 μL. The obtained RNA was processed in the reverse transcription reaction, which was performed using the *Enhanced Avian HS RT-PCR Kit* (Sigma-Aldrich, Poznan, Poland). The reaction mixture consisted of the following components: dNTPs (final concentration 1 mM), anchored oligo (dT)23 (final concentration 3.5 μM), 2 μL of 10× buffer, RNase inhibitor (20 U) and Enhanced Avian Reverse Transcriptase (RT; 20 U). The cDNA was used immediately or stored in −20 °C.

### 4.8. Qualitative Analysis

The reaction mixture used for PCR amplification consisted of a cDNA template, 0.5 μM of each primer, 10×AccuTaq Buffer, 0.5 U of AccuTaq LA DNA Polymerase Mix (Sigma Aldrich, Poznan, Poland), 0.2 mM of each dNTP, and water to a final volume of 20 μL. Every experiment included a negative control (sample without a cDNA template). The primer sequences were as follows: 5′AAC ACT CCT CTT CTC TCA AAG C3′ and 5′GGC TTG TTC TTC ACC ATG TTC3′ for the *3-hydroxy-3-methylglutaryl CoA reductase* gene (GenBank accession number: FJ755158.2); and 5′AAG CCC AAT CGA AGA GAG GTA3′ and: 5′ATC TTC TCC CTG TTG GCC TT3′ for the reference gene (*actin)* (GenBank accession number: KF699319.1). The expected sizes of the amplicons were 121 bp for *3-hydroxy-3-methylglutaryl CoA reductase* gene and 187 bp for the *actin* gene. The primers were designed using the OligoCalc server [63]. The conditions for the amplification for qualitative PCR were as follows: initial denaturation at 94 °C for two minutes, denaturation at 94 °C for 15 s, primer annealing at 59 °C for 45 s, extension at 72 °C for 45 s repeated in 35 cycles, and the final extension at 72 °C for 3 min. The PCR products were analyzed with a 100 bp DNA ladder on 1.2 % TBE-agarose gel (Bioline, London, UK). The electrophoresis was performed for one hour at a constant voltage of 90 V. The gel was stained with ethidium bromide, visualized under UV light, and photographed using MiniBIS Pro (DNR Bio-Imaging System Ltd., Neve Yamin, Israel). The obtained results confirmed the presence of PCR amplicons of expected size.

### 4.9. Quantitative Analysis by Real-Time PCR

The real-time PCR was performed on the corresponding cDNA synthesized from every sample using a Rotor-Gene 6000 (Corbett Research Pty Ltd., Mortlake, Australia). The investigated gene and a reference gene were amplified in parallel for each sample in separate wells, during the same PCR run. A reference gene was utilized as an internal positive control and as a normalizer for the correction of gene expression. For each PCR run, a reaction mixture was prepared consisting of 7.5 μL SYBR^®^ Green JumpStart™ Taq ReadyMix™ (Sigma Aldrich, Poznań, Poland), 0.7 μL forward primer (final concentration 0.2 μM), 0.7 μL reverse primer, 4.1 μL nuclease-free water, and 2.0 μL template cDNA. The thermal cycling conditions were as follows: initial denaturation step at 95 °C for two minutes, 35 cycles at 94 °C for 30 s, 59 °C for 30 s, and 72 °C for 30 s, and the final extension step at 72 °C for 3 min. After the reaction, a melting curve was performed to confirm reaction specificity. The experiments for all the samples were performed in triplicate. The 2^−ΔΔ*C*t^ method designed by Livak and Schmittgen [68] was used to calculate relative changes in *P. quinquefolium SSq* gene expression determined from the real-time quantitative PCR experiments. The real-time PCR assay has a 100% amplification efficiency for both genes and therefore it was used in the 2^−ΔΔ*C*t^ method [68]. The qPCR results were analyzed by Rotor-Gene 6000 Series Software version 1.7 (Qiagen, Hilden, Germany). The one-way ANOVA method with STATISTICA (StatSoft, Inc. 2011, version 10, www.statsoft.com) was applied to identify significant differences between tested samples. A *p*-value <0.05 was considered to be statistically significant [69].

### 4.10. Abscisic acid Treatment

Abscisic acid (ABA) (Sigma Aldrich, Poznan, Poland) was added to the modified Gamborg medium on day of inoculation or 25 day of culture when the *P. quinquefolium* hairy root culture was in its stationary growth phase. ABA was prepared as a stock solution, sterilized through a Millipore filter of pore size 0.20 μm (Merck Millipore Ltd., Burlington, MA, USA), and then added to the liquid media at final concentrations of 0 (control), 0.1, 0.25, 0.5, and 1 mg·L^−1^. The effect on the expression of the *HMGR* gene was examined after 6, 12, and 24 h of elicitor treatment. Biomass and ginsenoside accumulation in hairy root cultures of *P. quinquefolium* were measured after 1, 3, and 28 days of abscisic acid treatment.

### 4.11. HPLC Analysis of Ginsenosides

The *P. quinquefolium* cultures were harvested after one, three, and twenty-eight days of elicitation. Fresh hairy roots were dried at room temperature and processed for ginsenoside extraction and HPLC analysis as described previously [70].

All the tests were repeated in triplicate. The results concerning ginsenoside content were analyzed using the Kruskal–Wallis test with STATISTICA software (StatSoft, Inc. 2011, version 10, www.statsoft.com) and *p* < 0.05 was considered as statistically significant.

## 5. Conclusions

One of the cis-elements presented in the promoter of the *PqHMGR* gene is the ABRE motif, which responds to the effect of ABA. In silico analysis suggests that ABA may act on the expression of the *PqHMGR* gene and this was confirmed by RT-PCR analysis. In addition, ABA can regulate ginsenoside biosynthesis in hairy root cultures of *P. quinquefolium.* The longest exposure time (28 days) was characterized by the highest protopanaxatriol derivative (Rg1, Rg2, Re) production, but with depressed Rb saponin content compared to controls.
